# β-Cyclodextrin and oleic acid increase levels of dopamine and potentiates oxidative damage in young and adult rat brain

**DOI:** 10.1186/s12944-018-0816-3

**Published:** 2018-07-25

**Authors:** David Calderón Guzmán, Norma Osnaya Brizuela, Maribel Ortiz Herrera, Hugo Juárez Olguín, Armando Valenzuela Peraza, Gerardo Barragán Mejía

**Affiliations:** 1 Laboratorio de Neuroquímica, Instituto Nacional de Pediatría (INP), Mexico City, Mexico; 2 Laboratorio de Bacteriología Experimental, INP, Mexico City, Mexico; 3 Laboratorio de Farmacología, INP. Facultad de Medicina, Universidad Nacional Autónoma de México, Avenida Imán N° 1, 3rd piso Colonia Cuicuilco CP, 04530 Mexico City, Mexico

**Keywords:** Brain, Dopamine, Oleic acid, β-Cyclodextrin, Oxidative damage

## Abstract

**Background:**

Cyclodextrins are active pharmaceutical ingredients to treat neurological diseases by reducing neurotoxicity. The aim of this study was to test if combined consumption of β-cyclodextrin (BCD) and Oleic acid (OA) potentiates brain antioxidant protection.

**Methods:**

Four groups of young Wistar rats, grouped in 6 animals each, were treated as follows: Group (G) 1, saline solution 0.9% (control); G2, BCD (0.7 g/kg); G3, OA (15 ml/kg); G4, BCD + OA. The same design was assayed for groups of adult rats. Treatments were daily administered by oral means for five consecutive days. On the last day of administration, brains of the animals were extracted to measure dopamine, 5-HIAA, glutathione (GSH), ATPase, Lipoperoxidation and H_2_O_2._

**Results:**

Oleic acid and β-cyclodextrin upgraded the levels of dopamine, 5-HIAA and lipid peroxidation and downgraded the concentrations of GSH and H_2_O_2_ in cortex, hemispheres (striatum) and cerebellum/medulla oblongata regions.

**Conclusions:**

The results of the present study suggest that combined use of oleic acid and β-cyclodextrin may increase oxidative damage in brain regions and promote alteration in dopamine and 5-HIAA amines and hence, constitutes health risks among age of subjects.

## Background

Cyclodextrins (CDs) are a group of nontoxic oligosaccharides that is widely used as drug excipients and protein stabilizers. CDs have also been found to reduce the neurotoxicity [1], but also they are active pharmaceutical ingredients to treat neurological diseases [2]. Neurological disorders suggest increase in intracellular Ca^2+^ concentrations leading to an increase in the formation of reactive oxygen (ROS) and nitrogen (RNS) species by lethal pathways [3]. In neurodegenerative disorders, oxidative stress provokes mitochondrial alterations and DNA damage [4]. It has been found that brain aging is accompanied by a decrease in mitochondrial function [5]. However this damage can be treated with R-α-lipoic acid (R-LA) which is a cofactor of mitochondrial enzymes and a very strong antioxidant [6].

Nitric oxide is a good neuromodulator however an extra amount of it may lead to cell damage by oxidative stress or by decreasing reduced glutathione levels via nitroso-glutathione (NOGSH) formation within the cell [7]. Since free radicals are known to damage cell components [8], mainly plasma membrane lipids [9], the central nervous system (CNS) is particularly susceptible and extremely dependent on the amount of antioxidants, especially during development, when brain metabolism and growth rates are high [10], and regulates energy and glucose homeostasis by acting on hypothalamic neuro circuits and higher brain circuits such as the dopaminergic system [11]. Brain plasma membrane phospholipids are in close contact with structural proteins embedded in the lipid bilayer [12], through which the ionic interchange occurs by the action of Na^+^, K^+^ ATPase that stimulates Na^+^ and K^+^ flow [13]. The inhibition of Na^+^, K^+^ ATPase activity induces excitatory amino acids release within the CNS [14]. The purpose of the present study was to compare the combined effect of β-cyclodextrin (BCD) and oleic acid (OA) on selected oxidative stress markers and the levels of dopamine in brain regions of young and adult rat.

## Methods

Four groups of Wistar rats, grouped in 6 animals each, were treated as follows: Group (G) 1, saline solution 0.9% (control); G2, BCD (7 g/kg); G3, OA (15 ml/kg); G4, BCD + OA. The same design was assayed for groups of adult rats. Treatments were orally administered for 5 days. Animals were maintained in a mass air displacement room with a 12-h light: 12-h dark cycle at 22 ± 2 °C with a relative humidity of 50 ± 10%. Balanced food (Rodent diet 5001) and drinking water were given to the animals ad libitum. In the fifth day of the treatment, the rats were sacrificed by decapitation 60 min after receiving the last dose, and their brains were extracted and put in NaCl 0.9% at 4 °C, idem. Animal experiments were carried out under strict compliance with the Guidelines for Ethical Control and Supervision in the Care and Use of Animals and all experimental procedures were done following national and international rules.

The brains were dissected in cortex, hemispheres (striatum) and cerebellum/Medulla oblongata in 5 volumes of 0.05 M TRIS-HCl, pH 7.4 and used to evaluate ATPase activity and H_2_O_2_ level. An aliquot was obtained and homogenised in 0.1 M of perchloric acid (HClO_4_) (50:50 *v*/v), for the evaluation of reduced glutathione (GSH), dopamine, and 5-HIAA concentrations.

### Measurement of dopamine (DA)

The levels of DA were measured in the supernatant of tissue homogenized in HClO_4_ after centrifugation at 9000 rpm for 10 min in a microcentrifuge (Hettich Zentrifugen, model Mikro 12–42, Germany), with a version of the technique reported by Calderon et al. [15]. An aliquot of the HClO_4_ supernatant, and 1.9 mL of buffer (0.003 M octyl-sulphate, 0.035 M KH_2_PO_4_, 0.03 M citric acid, 0.001 M ascorbic acid), were placed in a test tube. The mixture was incubated for 5 min at room temperature in total darkness, and subsequently, the samples were read in a spectrofluorometer (Perkin Elmer LS 55, England) with 282 nm excitation and 315 nm emission lengths. FL Win Lab version 4.00.02 software was used. Values were calculated from a previously standardized curve and reported as μM/g of wet tissue.

### Measurement of 5-hydroxyindol acetic acid (5-HIAA)

5-HIAA levels were measured in the supernatant of tissue homogenized in HClO_4_ after centrifugation at 9000 rpm for 10 min in a microcentrifuge (Hettich Zentrifugen, model Mikro 12–42, Germany), with a modified version of the technique reported by Beck et al. [16]. An aliquot of the HClO_4_ supernatant and 1.9 ml of acetate buffer 0.01 M pH 5.5 were placed in a test tube. The mixture was incubated for 5 min at room temperature in total darkness, and subsequently, the samples were read in a spectrofluorometer (Perkin Elmer LS 55, England) with 296 nm excitation and 333 nm emission lengths. The FL Win Lab version 4.00.02 software was used. Values were inferred in a previously standardized curve and reported as nM/g of wet tissue.

### Measurement of reduced glutathione (GSH)

GSH levels were measured from the homogenised tissue of the supernatant of the perchloric acid obtained after centrifuging at 9000 rpm during 5 min (Mikro 12–42, Germany centrifuge) using a modified method of Hissin and Hilf [17]. 1.8 mL phosphate buffer pH 8.0 with EDTA 0.2%, 20 μL aliquot of the supernatant, and 100 mL of ortho-phthaldehyde (OPT) 1 mg/mL in methanol were poured in a test tube, and the mixture was incubated for 15 min at room temperature in absolute darkness. At the end of incubation, the samples were read spectrofluorometrically (PERKIN ELMER LS 55), with excitation and emission wavelengths of 350 and 420 nm respectively. FL Win Lab version 4.00.02 software was used. Values were inferred from a previously standardised curve and expressed as nM/g.

### Measurement of lipid peroxidation (Tbars)

Thiobarbituric acid reactive substances (Tbars) determination was carried out according to the method of Gutteridge and Halliwell [9], as follows: Each tissue sample was homogenized in 3 mL of phosphate buffer pH 7.4, from which 0.5 mL aliquot was taken and added to 1.5 mL of thiobarbituric acid (TBA) solution containing TBA (1.25 g), trichloroacetic acid (40 g) and concentrated HCl (6.25 mL) dissolved in 250 mL deionised water. The whole mixture was heated to water boiling point for 30 min (Thermomix 1420). Samples were then put in an ice bath for 5 min and then centrifuged at 3000 g for 15 min (Sorvall RC-5B Dupont). Supernatant absorbance was spectrophotometrically read in a three-set scheme at 532 nm (Heλios-α, UNICAM). The concentration of malondialdehyde (MDA) was calculated by the absorbance coefficient of MDA–TBA complex (1.56 X10^5^ M^− 1^ cm^− 1^). Tbars concentration was expressed as malondialdehyde μmoles per gram of wet tissue.

### Measurement of total ATPase

The activity of ATPase was assayed according to the method proposed by Calderón et al. [18]. 1 mg (10%) *w*/*v* of homogenised brain tissues in tris-HCl 0.05 M pH 7.4 was incubated for 15 min in a solution containing 3 mM MgCl_2_, 7 mM KCl, and 100 mM NaCl. To this was added 4 mM tris-ATP and incubated for another 30 min at 37 °C in a shaking water bath (Dubnoff Labconco). 100 μL 10% trichloroacetic acid *w*/*v* was used to stop the reaction and samples were centrifuged at 100 g for 5 min at 4 °C. Inorganic phosphate (Pi) was measured in triplicates using one supernatant aliquot as proposed by Fiske and Subarrow [19]. Supernatant absorbance was read at 660 nm in a Helios-α, UNICAM spectrophotometer and this absorbance was then expressed as mM Pi/g wet tissue per min.

### Measurement of H_2_O_2_

The determination of H_2_O_2_ was made using the modified technique of Asru [20]. Each brain region (cortex, striatum, cerebellum/medulla oblongata) was homogenized in 3 mL of tris-HCl 0.05 M pH 7.4 buffers. From the diluted homogenates, 100 μl was taken. 1 mL of potassium dichromate solution (K_2_Cr_2_O_7_) was added to the homogenates. The mixtures were heated to boiling point for 15 min (Thermomix 1420). The samples were later placed in an ice bath for 5 min and were centrifuged at 3000 g for 5 min (Sorvall RC-5B Dupont). The absorbances of the floating were read in triplicate at 570 nm in a spectrophotometer (Heλios-α of UNICAM). The concentration of H_2_O_2_ was expressed in μMoles.

### Statistical analysis

Analysis of Variance (ANOVA) or Kruskal-Wallis tests were used with their corresponding contrasts, and previous variance homogeneity comparison. Values restricted to *p* < 0.05, were considered statistically significant [21]. JMP Statistical Discovery from SAS version 8.0 software was used.

## Results

### General results

The statistical analysis of the data unveiled significant differences between the control group and the treated groups in all brain regions and for all the indicators. With the exception of GSH and H_2_O_2_ (Figs. 3 and 5) the concentrations of the different indicators were lower for both adult and young animals in the control group when compared with the experimental groups.

### Levels of 5HIAA

#### Adult animals

In the control group the concentration of 5HIAA was four times lower than those observed in the treated groups. On comparing the concentrations of the indicator between the experimental groups, it was observed that in the cortex there was a significant decrease in the group treated with the combination of cyclodextrin + oleic acid when compared with the group that received only oleic acid. In the hemispheres, the significant difference was between the groups that received administration of cyclodextrin and cyclodextrin + oleic acid. With regard to cerebellum/medulla oblongata region, a decrease in the concentration of HIAA was observed in the cyclodextrin + oleic acid group when compared with the groups that received only cyclodextrin or oleic acid.

#### Young animals

**Fig. 1:**
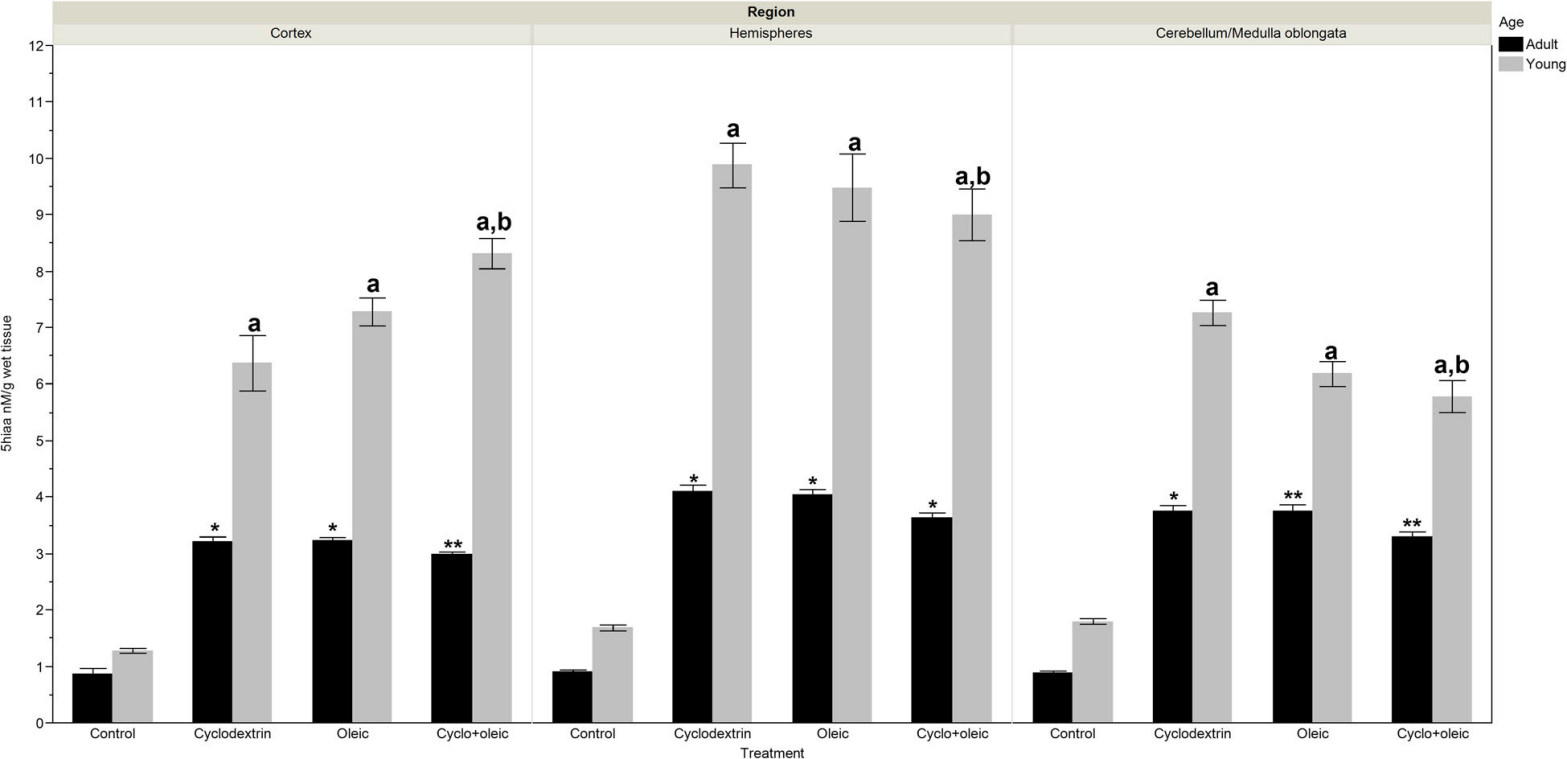
Levels of 5-hydroxyindol acetic acid (5-HIAA) in brain regions of adult and young Wistar rats treated with oleic acid and cyclodextrin. Adult rat. *Cortex*: Anova F = 240.29 *p* < 0.0001. *) *p* < 0.0001 vs control, **) *p* = 0.047 vs Oleic. *Hemispheres*: Kruskal-Wallis X^2^ = 41.57 *p* < 0.0001. *) *p* < 0.0001 vs control, **) *p* = 0.026 vs Cyclodextrin. *Cerebellum/ Medulla Oblongata*: Kruskal-Wallis X^2^ = 43.39 *p* < 0.0001. a) *p* < 0.0001 vs control, **) *p* = 0.013 vs Oleic and *p* = 0.009 vs Cyclodextrin Young rat. *Cortex*: Kruskal-Wallis X^2^ = 47.54 *p* < 0.0001. a) *p* < 0.0001 vs control, a,b) *p* = 0.039 vs Oleic, and *p* = 0.017 vs Cyclodextrin. *Hemispheres*: Kruskal-Wallis X^2^ = 42.07 *p* < 0.0001. a) *p* < 0.0001 vs control. *Cerebellum/medulla oblongata*: Kruskal-Wallis X^2^ = 49.75 *p* < 0.0001. a) *p* < 0.0001 vs control, a,b) *p* < 0.04 vs Cyclodextrin.

By comparing the concentrations of 5HIAA among the experimental groups, a significant increase was observed in the cortex of the group that received a combination of cyclodextrin + oleic acid with respect to animals treated with oleic acid or cyclodextrin. In the case of medulla oblongata this bioamine shows a decrease in the group administered with cyclodextrin + oleic acid when compared with the group to which only cyclodextrin was administered. However, an opposite effect was seen in the same brain region when this group was compared with the oleic acid group (Fig. 1).

### Levels of dopamine

#### Adult animals

The increase in the concentration of dopamine observed in the experimental groups was three times higher than what was seen in the control group. Comparison among the treated groups unveiled significantly lower level in the concentration of this bioamine in cyclodextrin + oleic group with regard to those treated with only oleic or cyclodextrin.

#### Young animals

**Fig. 2:**
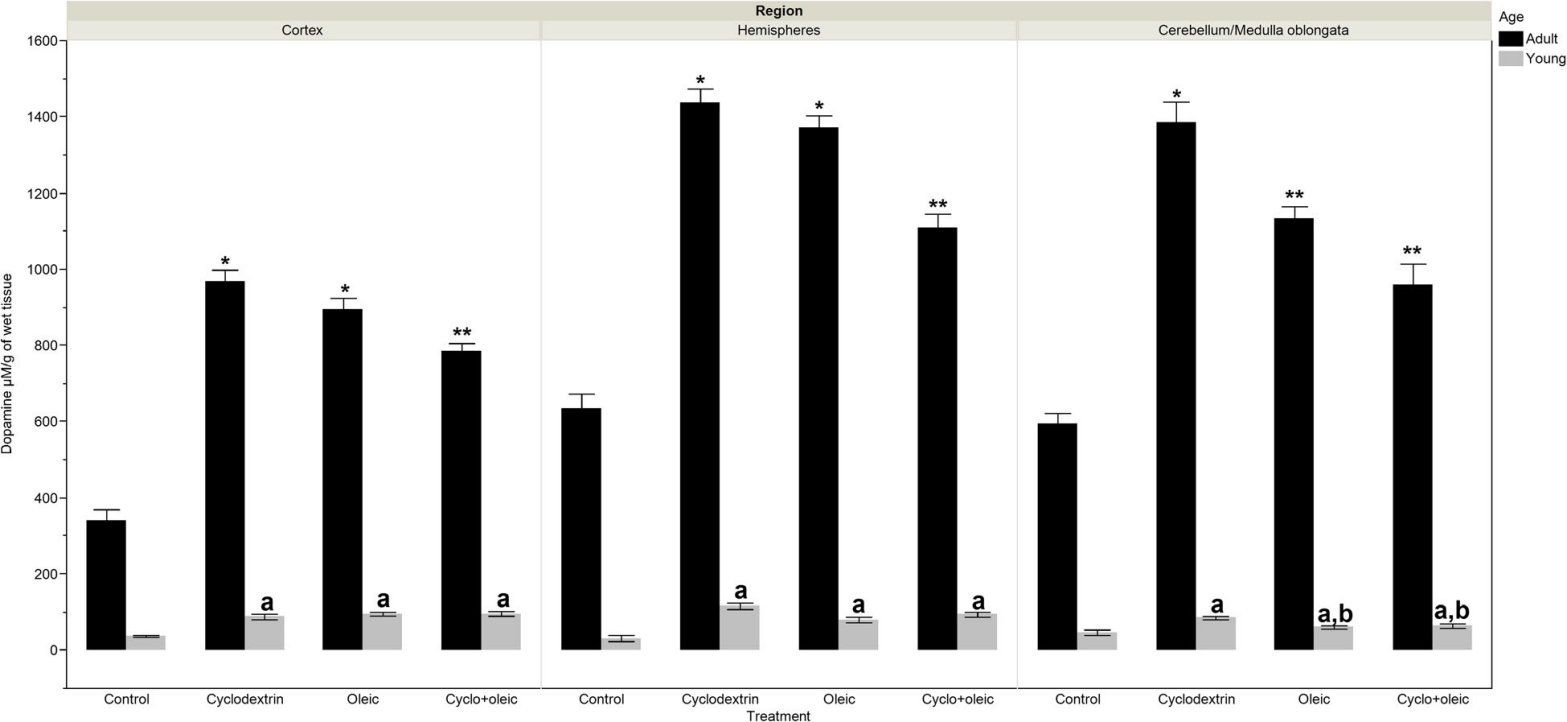
Levels of dopamine in brain regions of adult and young Wistar rats treated with oleic acid and cyclodextrin. Adult rat. *Cortex*: Anova F = 93.78 *p* < 0.0001. *) *p* < 0.0001 vs control, **) *p* < 0.01 vs Oleic y Cyclodextrin. *Hemispheres:* Anova F = 94.26 *p* < 0.0001 *) *p* < 0.0001 vs control, **) *p* < 0.0001 vs Oleic y Cyclodextrin. *Cerebellum/Medulla Oblongata*: Kruskal-Wallis X2 = 44.74 *p* < 0.0001. *) *p* < 0.0006 vs control, **) *p* < 0.004 vs Cyclodextrin. Young rat. *Cortex:* Kruskal-Wallis X^2^ = 34.26 *p* < 0.0001. a) *p* < 0.0006 vs control. *Hemispheres*: Anova F = 21.96 *p* < 0.0001. a) *p* < 0.0001 vs control, b) *p* = 0.005 vs Oleic. *Cerebellum/medulla oblongata*: Anova F = 8.35 *p* < 0.0001. a) *p* < 0.0001 vs control, a,b) *p* < 0.04 vs Cyclodextrin

With the exception of cerebellum/medulla oblongata, the levels of dopamine showed no differences among the experimental groups. In this region however, differences in the levels of this bioamine between the group treated with oleic acid and the control group was not observed. Nevertheless, when the group that received cyclodextrin is compared with the administration of oleic acid + cyclodextrin, there was an increase in the level of dopamine (Fig. 2).

### Levels of GSH

#### Adult animals

Glutathione concentration showed significantly higher level in the control group to the tune of 8-folds when compared with the experimental groups. In the cortex, the group with administration of cyclodextrin depicted a significantly lower concentration with respect to the group that received cyclodextrin + oleic acid. In hemispheres and cerebellum/medulla oblongata, the concentration of this indicator portrayed no significant differences.

#### Young animals

**Fig. 3:**
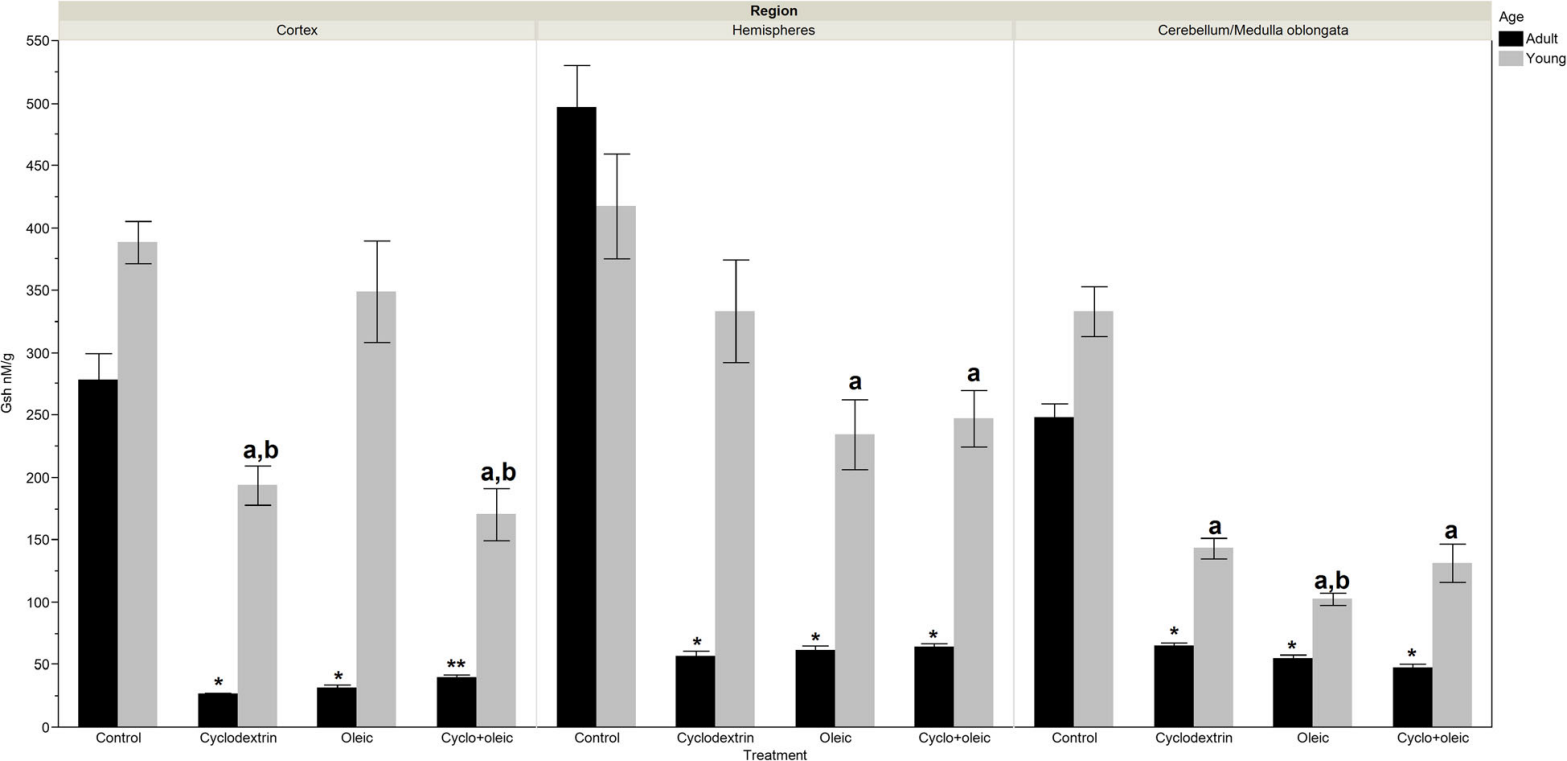
Levels of glutathion (GSH) in brain regions of adult and young Wistar rats treated with oleic acid and cyclodextrin. Adult rat. *Cortex*: Kruskal-Wallis X^2^ = 46.93 *p* < 0.0001. *) *p* < 0.0001 vs control, **) *p* < 0.047 vs Oleic y *p* = 0.0001 vs Cyclodextrin. *Hemispheres*:: Anova F = 200.18 *p* < 0.0001. *) *p* < 0.0001 vs control. *Cerebellum/Medulla Oblongata*: Anova F = 281.38 *p* < 0.0001. *) *p* < 0.0001 vs control. Young rat. *Cortex*: Kruskal-Wallis X^2^ = 36.72 *p* < 0.0001. a) *p* < 0.0001 vs control, a,b) *p* < 0.009 vs Oleic. *Hemispheres*: Kruskal-Wallis X^2^ = 17.12 *p* = 0.0007. a) *p* < 0.006 vs control. *Cerebellum/medulla oblongata*: Kruskal-Wallis X^2^ = 42.64 *p* < 0.0001. a) *p* < 0.0001 vs control, a,b) *p* = 0.0027 vs Cyclodextrin

The concentration of glutathione was higher in the control group with respect to the experimental groups. However, this was slightly significant in the cortex for the group treated with only cyclodextrin and cyclodextrin + oleic acid and in the hemisphere for the group that received oleic acid and cyclodextrin + oleic acid while in the cerebellum/medulla oblongata, the slight difference was seen in all the experimental groups. The comparison among the experimental groups revealed significant decrease of this indicator in cortex of the groups treated with only cyclodextrin and cyclodextrin + oleic acid with respect to the group that received only oleic acid. In the hemisphere, when the groups with oleic acid and cyclodextrin + oleic acid were contrasted with the group that received only cyclodextrin, the level of this indicator did not reach statistical difference (Fig. 3).

### Levels of Tbars

#### Adult animals

The increase in the levels of Tbars in all the experimental groups with respect to the control group had statistically significant differences. In the three regions and in cyclodextrin + oleic group, the concentration of Tbars resulted to be significantly less when compared with only oleic or cyclodextrin group.

#### Young animals

**Fig. 4:**
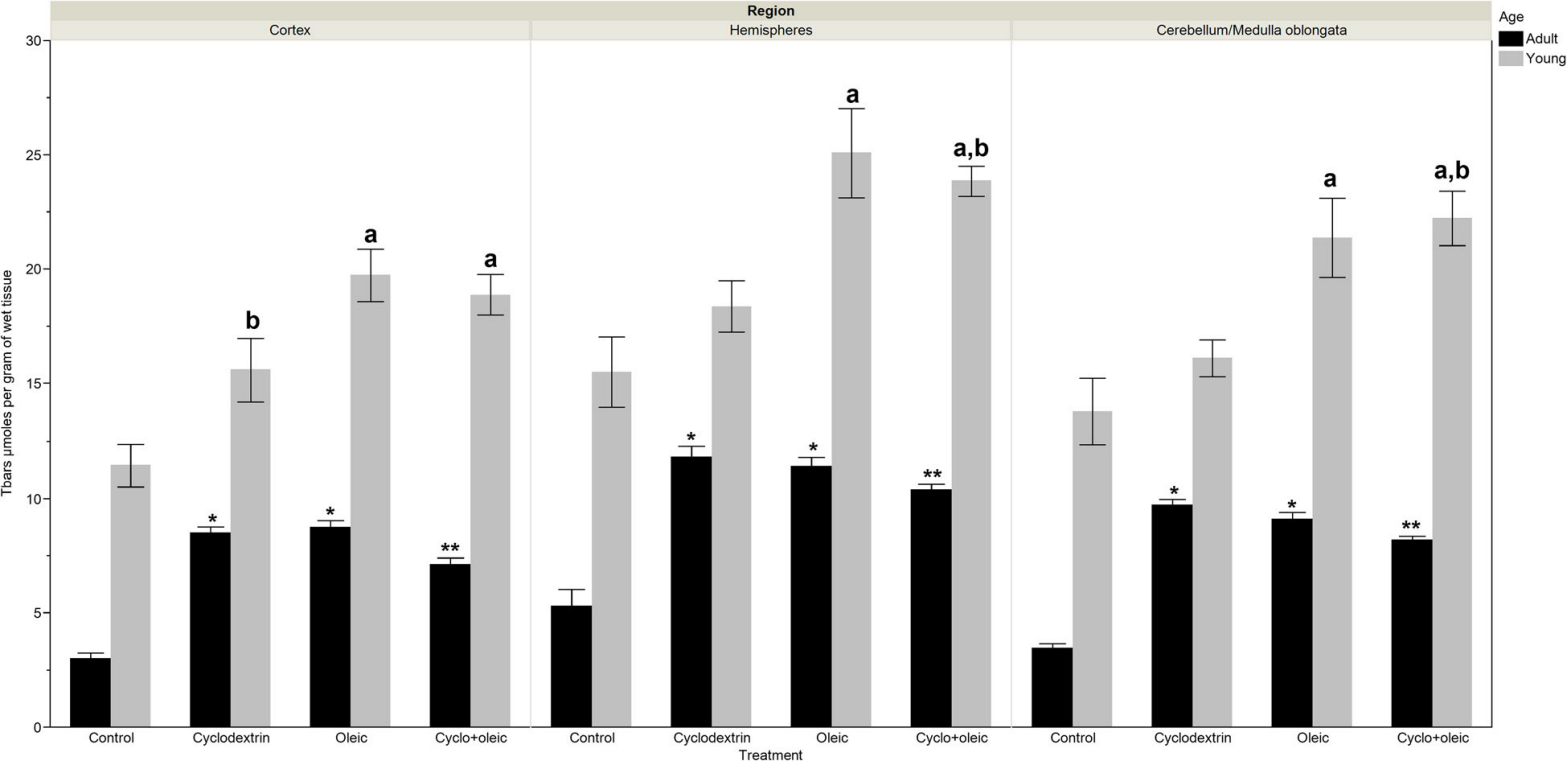
Levels of Tbars (lipid peroxidation) in brain regions of adult and young Wistar rats treated with oleic acid and cyclodextrin. Adult rat. *Cortex*: Anova F = 76.29 *p* < 0.0001. *) *p* < 0.0001 vs control, **) *p* = 0.0002 vs Oleic and *p* = 0069 vs Cyclodextrin. *Hemispheres*: Kruskal-Wallis X^2^ = 28.17 *p* < 0.0001. *) *p* < 0.0015 vs control, **) *p* = 0.002 vs Cyclodextrin. *Cerebellum/medulla oblongata*: Anova F = 115.37 *p* < 0.0001. *) *p* < 0.0001 vs control, **) *p* = 0.031 vs Oleic y *p* = 0.0003 vs Cyclodextrin. Young rat. *Cortex*: Anova F = 10.68 *p* < 0.0001. a) *p* < 0.0002 vs control, b) *p* = 0.04 vs Oleic. *Hemispheres*: Kruskal-Wallis X^2^ = 21.69 *p* < 0.0001. a) *p* < 0.01 vs control, b) *p* = 0.011 vs Cyclodextrin. *Cerebellum/medulla oblongata*: Kruskal-Wallis X^2^ = 22.63 *p* < 0.0001. a) *p* < 0.003 vs control, b) *p* = 0.003 vs Cyclodextrin

There is an increase in the levels of Tbars in all the experimental groups when compared with the control group. Nevertheless, this was statistically significant in the three brain regions for the groups treated with oleic acid and cyclodextrin. In the group that received only cyclodextrin, significant differences were not appreciated when compared with the control however, the concentration of Tbars resulted to be less significant when weighed against the group that received only oleic in the cortex and cyclodextrin + oleic in cerebellum/medulla oblongata (Fig. 4).

### Levels of H_2_O_2_

#### Adult animals

As was observed with GSH, the levels of H_2_O_2_ were significantly higher in the control group when compared with the experimental groups. In the cortex, the group with the administration of cyclodextrin + oleic displayed a significantly lower level with regard to the group that received only oleic. In the hemispheres, significant differences were not observed among the experimental groups. In cerebellum/medulla oblongata region, the levels of H_2_O_2_ were significantly higher in the group treated with only cyclodextrin in comparison with the group with administration of only oleic.

#### Young animals

**Fig. 5:**
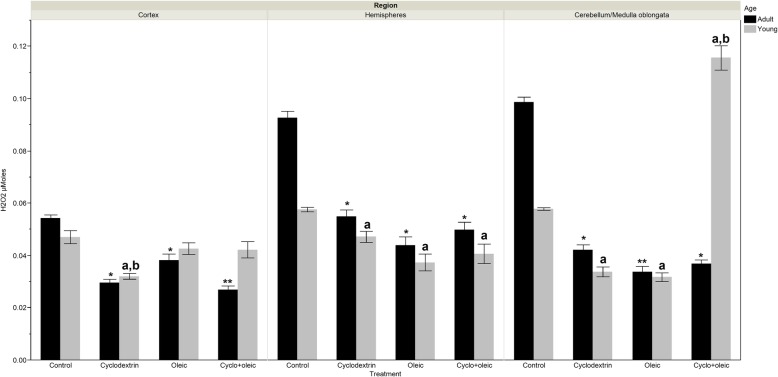
Levels of H_2_O_2_ in brain regions of adult and young Wistar rats treated with oleic acid and cyclodextrin. Adult rat. *Cortex*: Kruskal-Wallis X^2^ = 39.72 *p* < 0.0001. *) *p* < 0.0002 vs control, **) *p* = 0.0065 vs Oleic. *Hemispheres*: Kruskal-Wallis X^2^ = 39.32 *p* < 0.0001. *) *p* < 0.0001 vs control. *Cerebellum/ Medulla oblongata*: Anova F = 226.04 *p* < 0.0001. *) *p* < 0.0001 vs control, **) *p* = 0.018 vs Cyclodextrin. Young rat. *Cortex*: Kruskal-Wallis X^2^ = 21.99 *p* < 0.0001. a) *p* < 0.0001 vs control, b) *p* = 0.005 vs Oleic, and *p* = 0.033 vs Cyclodextrin. *Hemispheres*: Kruskal-Wallis X^2^ = 24.19 *p* < 0.0001. a) *p* < 0.009 vs control. *Cerebellum/medulla oblongata*: Kruskal-Wallis X^2^ = 63.34 *p* < 0.0001. a) *p* < 0.0001 vs control, b) *p* < 0.0001 vs Oleic and Cyclodextrin

As was observed with GSH, the levels of H_2_O_2_ were significantly higher in the control group with respect to the experimental groups although in the cortex, only the group treated with only cyclodextrin showed concentrations significantly less when compared with the control. Among the experimental groups, the levels of H_2_O_2_ in the cortex were significantly less in the group that received only cyclodextrin in comparison with the other groups. In cerebellum/medulla oblongata, the increase in H_2_O_2_ observed in the group with administration of cyclodextrin + oleic was thrice higher than what was seen in the other experimental groups and twice as that of control group (Fig. 5).

### Activity of ATPase

#### Adult animals

The activity of this enzyme showed a decrease in the cortex of animal groups that received cyclodextrin + oleic acid with respect to the control group and animal group treated with only oleic acid. On the contrary, an increase in the activity of the enzyme was seen in the cerebellum/medulla oblongata of the control and oleic groups while in hemisphere, no significant differences were observed.

#### Young animals

**Fig. 6:**
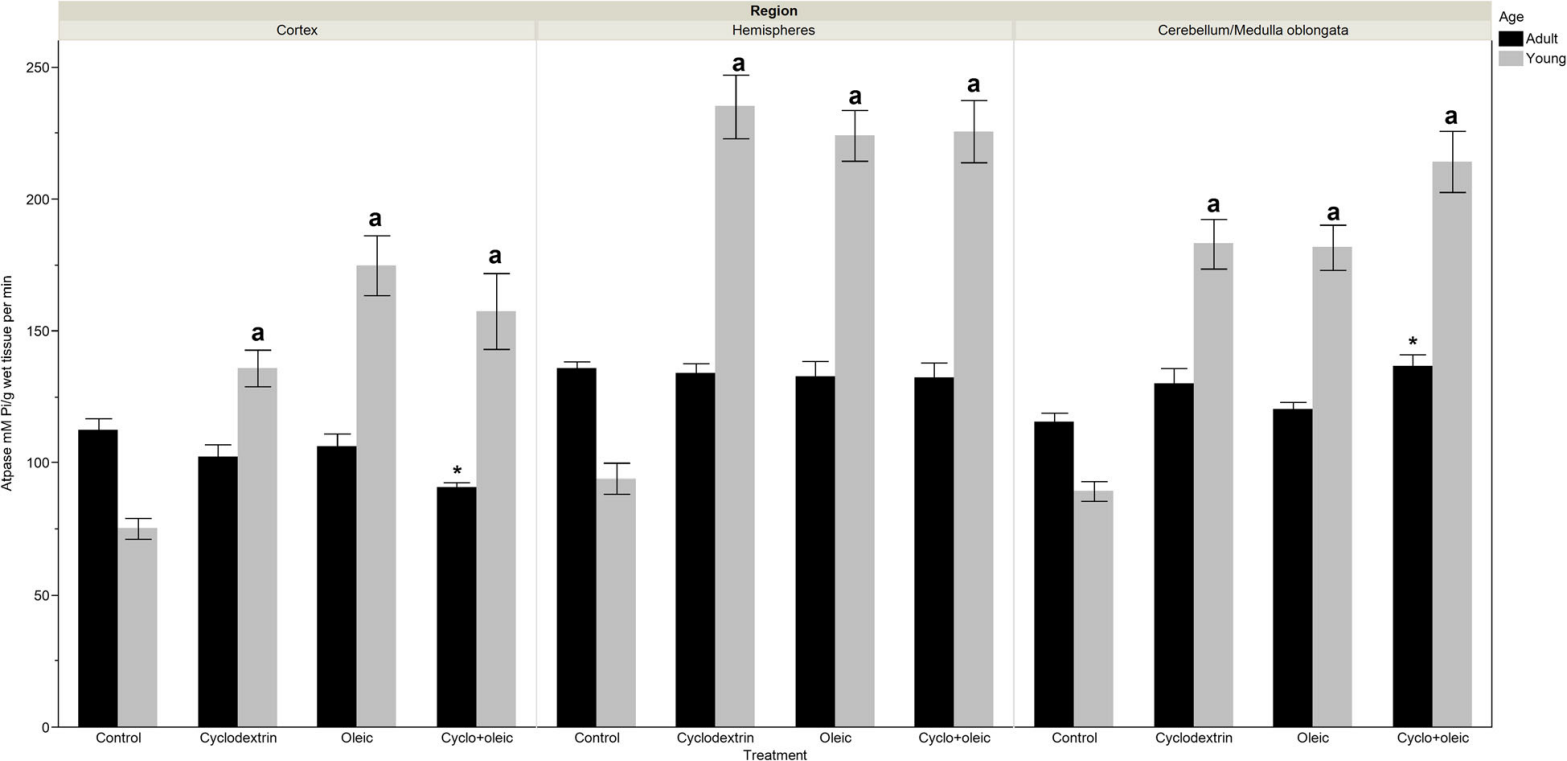
Levels of ATPase activity in brain regions of adult and young Wistar rats treated with oleic acid and cyclodextrin. Adult rat. *Cortex*: Kruskal-Wallis X^2^ = 18.55 *p* = 0.0003. *) *p* < 0.0002 vs control and *p* = 0.010 vs Oleic. *Hemispheres*: Kruskal-Wallis X^2^ = 2.63 *p* = 0.48. *Cerebellum/medulla oblongata*: Kruskal-Wallis X^2^ = 11.00 *p* < 0.011. a) *p* < 0.031 vs control and *p* = 0.026 vs Oleic. Young rat. *Cortex*: Kruskal-Wallis X^2^ = 38.08 *p* < 0.0001. a) *p* < 0.0001 vs control. *Hemispheres*: Kruskal-Wallis X^2^ = 39.84 *p* < 0.0001. a) *p* < 0.0001 vs control. *Cerebellum/medulla oblongata*: Kruskal-Wallis X^2^ = 43.09 *p* < 0.0001. a) *p* < 0.0001 vs control

The increase in the activity of this enzyme was twice bigger in the experimental groups when compared with the control. Among the groups that received the different treatments, significant differences were not observed (Fig. 6).

## Discussion

Methyl β-cyclodextrin, which promotes cholesterol depletion in cell membranes in brain [22], increased dopamine and 5-HIAA amines in brain of young rats while, compared with adult animals the concentration of 5HIAA decreases. This may be owed to the fact that changes in cholesterol concentration in the plasma membrane of presynaptic nerve terminals lead to nonspecific modulation of amine transports and homeostasis in the central nervous system [23].

CDs are a group of nontoxic oligosaccharides with 6 glucose subunits that are widely used as drug excipients and protein stabilizers. They come as a mixture of isomers with various degrees and pattern of hydroxypropylation [24]. Probably these isomers induced the increase of lipoperoxidation and lower levels of GSH in the brain regions and hence loss of cell viability for the inability to suppress oxidative stress. Also, CDs forms a hydrophobic central cavity that binds lipids which in animal studies and in previous clinical trials has been shown to alter plasma lipid levels [25], and membrane fluidity [26]. Moreover, such hydrophobic cavity has been linked with induction of an increase in the ATPase activity in cortex and hemisphere regions of young animals, and a decrease in adult animals. In fact, the oxidative damage was more evident in adult animals, probably because brain aging is accompanied by a decrease in mitochondrial function [5].

The present study was made in demand for evidence-based recommendations for the use of antioxidants in the prevention and treatment of pediatric ailments.

## Conclusion

The results suggest that the combination of high doses of oleic acid and β-cyclodextrin seems unsafe for healthy young and adult subjects for the fact that it induces an increase in dopamine and 5-HIAA amines. Oxidative damage may be the end result of this effect.

## Abbreviations

5-HIAA: 5-hydroxyindoleacetic acid
BCD: β-cyclodextrin
CDs: Cyclodextrins
CNS: Central nervous system
DNA: Deoxyribonucleic acid
GSH: Glutathione
HClO4: Perchloric acid
NOGSH: Nitroso-glutathione
OA: Oleic acid
R-LA: R-α-lipoic acid
RNS: Reactive nitrogen
ROS: Reactive oxygen
Tbars: Thiobarbituric acid reactive substances
